# Robotic vs laparoscopic approach for single anastomosis duodenal-ileal bypass with sleeve gastrectomy: a propensity score matching analysis

**DOI:** 10.1007/s13304-022-01381-8

**Published:** 2022-09-25

**Authors:** Francesco Pennestrì, Luca Sessa, Francesca Prioli, Pierpaolo Gallucci, Luigi Ciccoritti, Francesco Greco, Carmela De Crea, Marco Raffaelli

**Affiliations:** 1 U.O.C. Chirurgia Endocrina E Metabolica, Centro Dipartimentale Di Chirurgia Endocrina E Dell’Obesità, Fondazione Policlinico Universitario Agostino Gemelli IRCCS, L.go A. Gemelli 8, 00168 Rome, Italy; 2 Centro Malattie Endocrine E Obesità, Fondazione Gemelli Giglio Cefalù, Cefalù, Palermo, Italia; 3 Centro di Ricerca in Chirurgia delle Ghiandole Endocrine e dell’Obesità, Università Cattolica del Sacro Cuore, Rome, Italy

**Keywords:** Single anastomosis duodeno-ileal bypass with sleeve gastrectomy, Laparoscopic surgery, Robotic surgery, Bariatric surgery, SADI-S, SADI

## Abstract

Biliopancreatic diversion with duodenal switch and single anastomosis duodenal-ileal bypass with sleeve gastrectomy (SADI-S) are technically demanding hypo-absorptive bariatric procedures. They are often indicated in superobese patients (BMI ≥ 50 kg/m^2^), as robotic platform could improve ergonomics against a thick abdominal wall, preventing bending of instruments and simplifying hand-sewn anastomoses. We aimed to report our experience with robotic SADI-S (R-group) and to compare outcomes with the laparoscopic (L-group) approach. Among 2143 patients who underwent bariatric procedures at our institution between July 2016 and June 2021, 116 (5.4%) consenting patients were scheduled for SADI-S as primary or revisional procedure: 94 L-group, 22 R-group. R-group and L-group patients were matched using PSM analysis to overcome patients selection bias. Postoperative complications, operative time (OT), post-operative stay (POS) and follow-up data were compared. After PSM, 44 patients (22 patients for each group) were compared (Chi-square 0.317, *p* = 0.985). Median age, gender, median BMI, preoperative rates of comorbidities, previous abdominal bariatric and non-bariatric surgeries and type of surgical procedures (SADI-S/SADI) were comparable. Median OT was shorter in the L-group (130 Vs 191 min, *p* < 0.001). 30-days’ re-operative complications and late complications rates were comparable. At 25-months’ mean follow-up, the median Percentage Excess Weight Loss (72%) was comparable between the groups (*p* = 0.989). L-group and R-group were comparable in terms of re-operative complication rate and short-term outcomes. The robotic platform may increase the rate of single step procedure in challenging cases. Larger studies with longer follow-up and cost-analysis are necessary to draw definitive conclusions.

## Introduction

The biliopancreatic diversion procedure with duodenal switch BPD-DS has been demonstrated to provide significantly greater weight loss than other bariatric procedures with concurrent sustained improvement in metabolic health in long-term studies [1]. However, BPD-DS should be considered a technically demanding operation. Complications are infrequent, but if a suture leak, a post-operative bleeding or an intestinal obstruction is present, the consequences for the patient might be severe, ranging from a simple prolongation of the hospital stay to sepsis, peritonitis, reoperations or even death [2].

With the intent to simplify the surgical procedure, reduce the operative time, decrease the potential complication rate and to maintain the outcome of the original procedure (BPD-DS), Sánchez-Pernaute and Torres and coworkers introduced in the clinical practice in 2007 a novel operation [3], in which only one anastomosis is performed, the “Single Anastomosis Duodeno-Ileal Bypass with Sleeve Gastrectomy” or SADI-S [4]. After initial promising results [3], the technique became popular all over the world [5]. Single-Anastomosis Duodeno-Ileal Bypass can be used as revisional surgery after failed Sleeve Gastrectomy or as planned second-stage surgery (after Sleeve Gastrectomy) (SADI) [6]. SADI-S can also be used as revisional surgery after failed adjustable gastric banding and gastric bypasses [7, 8]. Based on retrospective and multicentric studies, SADI-S is effective in achieving good initial loss and weight maintenance with acceptable long-term “nutritional complications” [4, 5, 9–11].

Conventional laparoscopy comes with some technical limitations, which are amplified by the difficulties accompanying obese patients in general and super-obese patients in particular. In terms of surgical challenges, these limitations include space constraints (often caused by increased liver size), intra-abdominal fat and a thick abdominal wall, which increases the level of difficulty in handling manual instruments used in minimally invasive surgery [1, 12]. Furthermore, current literature shows that the overall complication rate of laparoscopic bariatric procedures is as great as 20% and leak rates may reach 5.1% [13]. As a result patients may experience prolonged hospital stays, reoperations and even life-threatening complications. Thus, it is evident that improvements in clinical outcomes driven by advanced technologies in bariatric surgery are needed, especially in this special population [14]. Advantages of using the robotic system include greater dexterity and precision in tissue manipulation, especially in challenging cases and in anatomical regions that are difficult to access: this may result in fewer conversion rates [15] and probably fewer short-term complications [16]. SADI-S is a malabsorptive/hypoabsorptive challenging multi-quadrant procedure and is mostly indicated in the treatment of “complex” bariatric patients (BMI ≥ 50 kg/m^2^, metabolic patients and revisional surgery), so it is clear that it is the ideal condition to benefit from robotic technologies. Nevertheless, only anecdotic experience of robotic SADI-S are reported in the literature [17–20].

Therefore, the aim of this retrospective case–control study is to analyze our experience with robotic SADI-S and more particularly compare the outcomes of laparoscopic versus robotic approach using propensity score matching analysis to reduce selection biases.

## Methods

We conducted a retrospective review of prospectively collected data into a de-identified dedicated bariatric database of patients who underwent bariatric surgery (primary and revisional), between July 2016 and June 2021 at our tertiary referral center for bariatric and metabolic surgery, Center of Excellence of the Italian Society of Bariatric Surgery (SICOb).

**Study end-points.** The primary endpoint was to compare the robotic Vs laparoscopic approach for SADI-s in terms of complication rate. The secondary endpoint was to compare the two population (robotic Vs laparoscopic SADI-S) in terms of operative time, and postoperative hospital stay. Additionally, the learning curve of robotic Vs laparoscopic SADI-S was evaluated.

**Patients’ population.** All the patients who underwent SADI-S between July 2016 and June 2021 were identified among 2143 patients who underwent bariatric surgical procedures.

Patients who underwent robotic and laparoscopic SADI-S were identified and compared. The propensity score matching (PSM) was used to randomize. Propensity score matching was obtained with the “1:1 nearest neighbor” matching method (discard = both groups, caliper = 0.2). Type of surgical approach (laparoscopic or robotic) were entered into the regression model of the propensity score as the binary treatment variable. The covariates supposed to affect the challenging of surgical procedure were included into the analysis: age, gender, BMI, type of surgical procedures and previously not-bariatric abdominal surgery. Baseline characteristic, operative and post-operative variables were compared using a bivariate analysis. The presence of a normal distribution was assessed using the Shapiro–Wilks test. Fisher’s exact test and Chi-square test was used to compare categorical variables. Continuous variables were expressed as median (interquartile range, IQR). We used *t* test or Mann–Whitney test to compare continuous variables, depending on data distribution of the analyzed population.

To define the learning curve of robotic, laparoscopic, primary and revisional surgery, we used the Cumulative sum (CUSUM) analysis, as reported by other authors [21].

For this study, follow-up was closed on 31th July 2021.

Patients included in this study met the consensus criteria for bariatric surgery, fulfilled the national guidelines of SICOb [https://www.sicob.org/00_materiali/linee_guida_2016.pdf] and underwent primary or revisional single anastomosis duodeno-ileal bypass with sleeve gastrectomy by laparoscopic or robotic approach. Patients were fully informed of the surgical technique, anesthesia, effects and complications. Multidisciplinary bariatric evaluation (a team consisting of a surgeon, an endocrinologist, a dietician and a psychologist) was performed for each patient, to have a personalized bariatric process. The preoperative workup consisted of upper endoscopy, ultrasound of the abdomen, upper gastrointestinal (UGI) contrast study, blood analysis, respiratory investigation, nutritional status appraisal, psychological and cardiac evaluation.

**Definition.** The operative time is defined as the interval from incision to wound closure. Docking time is the time needed to place the robotic platform. Mortality was defined as any intraoperative or post-operative death within 30th-days from surgery. With the aim of reducing the errors in the interpretation of acronyms (SADI-S, SADI), during the discussion, we will use the term “SADIS” to identify both primary procedures and revisional ones.

### Patients’ selection for SADIS

o In our clinical practice, candidates for SADIS as a primary procedure are super-obese patients (BMI ≥ 50 kg/m^2^), especially with binge-eating habit and
p Metabolic patients
  Revisional procedures:
q   Two-step procedure in young patients (age < 40 years) and/or challenging cases (BMI ≥ 60 kg/m^2^, previously major abdominal surgery)
r   Inadequate weight loss and/or weight regain after sleeve gastrectomy and in other selected cases (previously laparoscopic adjustable gastric-banding, Roux-en-Y gastric bypass, one-anastomosis gastric bypass)

Absolute contraindications are:

- Barrett’s esophagus

- Severe gastro-esophageal reflux disease

- Major (> 4 cm) hiatal hernia

Indications for robotic approach: we used the robotic approach for challenging cases. “Challenging cases” are a clinical features not evaluable by a single parameter, indeed, it is determined by the occurrence of one or more of these conditions: patients with BMI ≥ 55 kg/m^2^, especially if males and/or with previously major abdominal surgery.

### Surgical techniques

All procedures were performed by the same expert bariatric surgeon, who performed more than 1000 laparoscopic bariatric procedures and more than 50 robotic bariatric procedures prior to performing the first case of robotic SADI-S in 2016.

### Laparoscopic single anastomosis duodeno-ileal bypass with sleeve gastrectomy

Patient lies in supine position legs open. A 5 mm optical trocar is inserted in left flank along the mid-clavicular line and pneumoperitoneum (14 mmHg) is made. Under visual control, one 12 mm trocar in upper umbilical region and two 5 mm trocars, respectively, in epigastrium and right hypochondrium, are placed. Procedure begins with the dissection of the gastrocolic ligament and proceeds with the preparation of the greater curvature, which is carried out cranially until the left diaphragm pillar is exposed and caudally to the pylorus. Then, preparation of the first part of the duodenum is performed, until gastroduodenal artery is exposed. The next step is the vertical gastric resection, which is performed using a laparoscopic linear stapler and sized upon a 40 F orogastric bougie. At this point, the first part of the duodenum is sectioned, approximately 2 cm after the pylorus, using a linear stapler. Next, caecum and last ileal loop are identified and 300 cm from ileocecal valve are counted: this is the point where gastro-ileal anastomosis will be made. The intestinal measurement is performed after infusion of 20 mg of Buscopan®, to get the maximum possible relaxation of the smooth muscle and perform the most accurate calculation of the common limb’s length. The selected loop is then anchored to the proximal sectioned duodenum with a PDS 3.0 stitch. At this point, a double-layer manual termino-lateral antecolic duodeno-ileal anastomosis between the sectioned proximal duodenum and the previously identified ileal loop is made, using PDS 3.0 for the external anterior layer and Stratafix 2.0 for the external posterior and the internal layer. At the end of the reconstructive phase, integrity of the anastomosis is verified with a blue methylene and pneumatic test. Sectioned stomach is then extracted through left flank trocar site and sent for histological examination. Hemostasis is verified and a 19 F drainage is placed behind the anastomosis trough the left flank trocar site. Finally, fascial and skin closure is performed.

### Robotic single anastomosis duodeno-ileal bypass with sleeve gastrectomy

Patient lies in supine position legs open. A 12 mm optical trocar is inserted in supraumbilical region and pneumoperitoneum (14 mmHg) is made. Under visual control, other four 8 mm robotic trocars are placed along a horizontal line cranially to the 12-mm trocar, in right and left hypochondrium and right and left paramedian region. Then, the caecum, the last ileal loop and the ileal loop where anastomosis will be made (at 300 cm from ileocecal valve) are identified. The selected loop is then inked and anchored to the omentum with a Vicryl 3.0 stitch. At this point, robotic docking is performed and the procedure follows the same above-mentioned steps.

### Laparoscopic/robotic single anastomosis duodeno-ileal bypass (revisional procedures after sleeve gastrectomy)

These procedures have the same steps described above. Obviously gastric resection is not carried out, because it is already performed, so surgery starts directly with the preparation of the duodenum.

### Post-operative protocol

A standard postoperative protocol personalized for bariatric patients was used. All patients remained nil per os until UGI contrast study was performed on postoperative day (POD) 1, as previously described [22]. UGI contrast studies were performed with water-soluble contrast (Gastrografin®, Bracco SpA, Milan, Italy). Liquid diet commenced after the UGI contrast study, if no leak was observed, and if clinical course was uneventful. Routine complete blood examination and blood count were obtained on POD1 in all patients. Further personalized examinations were obtained based on the clinical aspects of each patient. For example, patients with symptoms and signs of suspected leak despite negative UGI contrast had indication for further evaluation [abdominal computed tomography (CT) with intravenous ad oral contrast material and/or surgical exploration]. The severity of postoperative complications was rated according to the Clavien–Dindo classification [23]. Routine follow-up with blood test analysis and physical examination were performed on POD 30, then every 3 months for the first year, every 6 months for the second year and then annually, according to the SICOb guidelines [https://www.sicob.org/00_materiali/linee_guida_2016.pdf]. At discharge, patients were advised to follow a strict dietary regimen which consists of three progressive phases (liquid, semisolid and solid diet), each one of at least 2–3 weeks, with proteic, vitaminic and minerals supplementations. Proteic supplementation (Protifar, Nutricia, Milan, Italy, 55 g per day during the first dietary’s phase and 15 g per day during the second one) is indicated, because clinical practice guidelines for perioperative support of bariatric patients by the Endocrine and Obesity Societies recommend a daily intake of protein from a minimum of 60 up to 1.5 g/kg ideal body weight [24]. All the patients received FitForMe WLS Maximum® as vitamins and minerals supplementation. FitForMe WLS Maximum® is a customized multivitamin supplement for bariatric patients who underwent malabsorptive/hypoabsorptive procedures and contains elevated doses of multiple vitamins and minerals (see https://fitforme.it/product/pacchetto-wls-maximum/#1586345186042-d5da1ce5-60ed for details of composition). FitForMe WLS Maximum® is dosed as one capsule per day. All patients received enoxaparin (4000 UI/0.4 ml) for 4 weeks and proton-pump inhibitor (PPI) (esomeprazole, 40 mg daily) for at least 6 months, as part of the standard postoperative protocol.

### Discharge criteria

The discharge is scheduled 24 h after the surgical procedure whether the following conditions are satisfied: no clinical complications or postoperative biochemical and imaging alterations occurred; oral alimentation is tolerated; autonomy in life activities is acquired; the discharge is accepted by the patient.

### Statistical analysis

Basic demographic and clinical data were collected through review of patient charts and electronic databases. Other parameters, such as postoperative pain (pain scale 0–10), nausea, vomiting, drain output, urine output, hemoglobin level, white blood cells level, need for blood transfusion and operative findings (when further surgery was needed), were also registered.

Statistical analysis and PSM was conducted with SPSS 22.0 software for Windows (SPSS Inc, Chicago, III). STATA Version 17 (StataCorp LP, College Station, TX, USA) is used to perform CUSUM analysis. A value of < 0.05 was considered statistically significant.

## Results

During the study period, over 2143 bariatric procedures were performed (1915 primary procedures and 228 revisional procedures). A total of 116 patients were scheduled for single anastomosis duodeno-ileal bypass with sleeve gastrectomy: 85 (73.3%) for primary procedure and 31 (26.7%) for revisional one. The population’s characteristics are shown in Table 1. Laparoscopic approach was performed in 94 (81%) cases, while robotic approach was performed in 22 (19%) cases. Overall, the median preoperative BMI was 52.25 (7.7) Kg/m^2^, the median operative time was 120 (59) min and the median post-operative hospital stay was 2 (2) days. In this series, no other intra-abdominal procedures (such as cholecystectomy) have been performed and no intraoperative complications were reported. No intraoperative leaks were detected at the methylene blue test. No intraoperative deaths occurred. No conversions were required, either from laparoscopic to open surgery, or from robotic to laparoscopic/open surgery. No 30th-days mortality was registered. We reported 30th-day post-operative complications in 4 (3.4%) patients: one severe acute necrotizing pancreatitis of unknown origin (not biliary) that needed further surgery (Clavien–Dindo IV), one sub-ileus due to trocar site incisional hernia which needed reoperation (Clavien–Dindo IIIb), one health care acquired pneumonia (Clavien–Dindo II) and one late hematic collection near to the stomach suture line, without evidence of fistula (Clavien–Dindo II).

**Table 1:** Characteristics of study’s population

Patients	116
Age (years)	44 (13)
Height (cm)	168 (14)
Weight (kg)	141 (38.5)
BMI (kg/m^2^)	52.25 (7.7)
Male/female	40 (34.5%)/76 (65.5%)
Smoking	
No	74 (63.8%)
Previously (≥ 6 months)	24 (20.7%)
Previously (< 6 months)	18 (15.5%)
Comorbidities (yes/no)	70 (60.3%)/46 (39.7%)
HBP (yes/no)	45 (38.8%)/71 (61.2%)
OSAS (yes/no)	38 (32.8%)/78 (67.2%)
Diabetes	
No	89 (76.7%)
IGT	13 (11.2%)
DMT2	14 (12.1%)
Previous non-bariatric abdominal surgery	
No	56 (48.3%)
Laparoscopic	17 (14.7%)
Open	43 (37.1%)
Previous bariatric procedures	
No	78 (67.3%)
Sleeve gastrectomy	31 (26.7%)
Gastric banding	6 (5.2%)
Functional gastric bypass with an adjustable gastric banding	1 (0.8%)
BMI (kg/m^2^) at the first bariatric procedure	51.3 (12.6)
Procedure	
SADI-S	85 (73.7%)
SADI	31 (26.7%)
Operative time (minutes)	120 (59)
Surgical Approach	
Laparoscopic	94 (81%)
Robotic	22 (19%)
Intensive care unit (yes/no)	2 (1.4%)/114 (98.6%)
Postoperative hospital stay (days)	2 (2)
Postoperative 30th day complications (yes/no)	4 (3.4%)/112 (96.6%)
Reoperation (yes/no)	2 (1.7%)/114 (98.3%)
Pneumonia (yes/no)	2 (1.7%)/114 (98.3%)
Bleeding (yes/no)	1 (0.8%)/115 (99.2%)
Acute Pancreatitis (yes/no)	1 (0.8%)/115 (99.2%)
Trocar site hernia (yes)	1 (0.8%)/115 (99.2%)
Other postoperative 30th day complications: DVT, PE, AF, AMI, sleeve leakage, duodenal stump leakage, duodeno-ileal anastomosis leakage, small bowel perforation, sleeve stenosis, duodeno-ileal anastomosis stenosis, small bowel twisting, internal hernia	–
30th day hospital readmissions (yes/no)	1 (0.8%)/115 (99.2%)
Late complications (yes/no)	4 (3.4%)/112 (96.6%)
Incisional hernia (yes/no)	1 (0.8%)/115 (99.2%)
Chronic diarrhea (yes/no)	3 (2.6%)/112 (97.3%)
Malnutrition (yes/no)	2 (1.7%)/114 (98.3%)
Other late complications: sleeve stenosis, duodeno-ileal anastomosis stenosis, small bowel twisting, internal hernia, duodeno-ileal anastomosis ulcer, bile reflux	–
Other events (yes/no)	
Wernike–Korsakoff’s syndrome	1 (0.8%)/115 (99.2%)
Toxic megacolon	1 (0.8%)/115 (99.2%)
Follow-up time (months)	25 (25)
%EWL	72 (44)

*BMI* body mass index, *HBP* high blood pressure, *OSAS* obstructive sleep apnea syndrome, *IGT* impaired glucose tolerance, *T2DM* type 2 diabetes mellitus, *DVT* deep vein thrombosis, *PE* pulmonary embolism, *AF* Atrial Fibrillation, *AMI* acute myocardial infraction, *SADI-S* single anastomosis duodeno-ileal bypass with sleeve gastrectomy, *SADI* single anastomosis duodeno-ileal bypass in previously sleeve gastrectomy, *%EWL* percentage of excess weight loss

At median follow-up time of 25 (25) months, the median %EWL (percentage of excess weight loss) was 72% (44), the median BMI was 30 (10.85) Kg/m^2^, and the median daily bowel movements was 3 (1).

We divided the population in two groups according to the surgical approach used: laparoscopic group (L-group) and robotic group (R-group). Table 2 reports the characteristics of the two groups. L-groups and R-group were comparable for demographics and preoperative variables.

**Table 2:** Data and statistical analysis of the laparoscopic-group and robotic-group

	Laparoscopic-group	Robotic-group	*p*
Patients	94	22	–
Age (years)*	44.5 (12)	42.0 (13)	0.988
Height (cm)*	168.0 (12)	169.0 (19)	0.740
Weight (kg)*	140.0 (37,5)	158.5 (34.8)	0.053
BMI (kg/m^2^)°	52.05 (10.1)	53.45 (6.8)	0.078
Male/female	30/64	10/12	0.319
Smoking			
No	58	16	
Previously (≥ 6 months)	10	4	0.564
Previously (< 6 months)	16	2	
Comorbidities (yes/no)	58/36	12/10	0.630
HBP (yes/no)	38/56	7/15	0.628
OSAS (yes/no)	32/62	6/16	0.621
Diabetes			
No	71	18	
IGT	10	3	0.470
DMT2	13	1	
Previous non-bariatric abdominal surgery			
No	45	18	
Laparoscopic	14	3	0.470
Open	35	1	
Previous bariatric procedure			
No	58		
Sleeve gastrectomy	30	1	
Gastric banding	5	1	0.102
Functional gastric bypass with an adjustable gastric banding	1		
BMI (kg/m^2^) at the first bariatric procedure	50.4 (12.6)	56.85	–
Procedure			
SADI-S	64	21	0.007
SADI	30	1	
Operative time (minutes)°	120.0 (49)	191.54 (49)	< 0.001
Docking time (minutes)	–	10.5 (8)	–
Intensive care unit (yes/no)	1/93	1/21	0.345
Postoperative hospital stay (days)°	3 (2)	2 (1)	0.323
Postoperative 30th day complications (yes/no)	1/93	3/19	0.021
Reoperation (yes/no)	1/93	1/21	0.345
Pneumonia (yes/no)	1/93	1/21	0.345
Bleeding (yes/no)	0/94	1/22	0.190
Acute pancreatitis (yes/no)	1/93	0/22	1
Trocar site hernia (yes/no)	0/94	1/21	0.190
30th day hospital readmissions (yes/no)	0/94	1/22	0.345
Late complications (yes/no)	3/91	0/22	1
Incisional hernia (yes/no)	1/93	0/22	1
Chronic diarrhea (yes/no)	3/91	0/22	1
Malnutrition (yes/no)	2/92	0/22	1
Other events (yes/no)			
Wernike–Korsakoff’s syndrome	1/93	0/22	1
Toxic megacolon	1/93	0/22	1
Follow-up time (months)	27 (25)	6 (23)	< 0.001
%EWL	75.45 (46)	67.1 (42)	0.989

*BMI* body mass index, *HBP* high blood pressure, *OSAS* obstructive sleep apnea syndrome, *IGT* impaired glucose tolerance, *T2DM* type 2 diabetes mellitus, *SADI-S* single anastomosis duodeno-ileal bypass with sleeve gastrectomy, *SADI* single anastomosis duodeno-ileal bypass in previously sleeve gastrectomy, *%EWL* percentage of excess weight loss

^*^Student’s *t* test

°Mann–Whitney *U* test

After PSM, the study population was composed by 44 patients: 22 in L-group and 22 in R-group (overall balance test: chisquare 0.371, *p* = 0.985; multivariate imbalance measure L1 after matching 0.227). All data are reported in Table 3. Patients were comparable for age, gender, weight, preoperative BMI, rates of comorbidities and previous not-bariatric abdominal surgery. The median operative time was significantly shorter for L-group: 130 (47) vs 191.54 (49) minutes (*p* < 0.001). The median docking time for robotic procedures was 10.5 (8) min. Even if we exclude, for the R-group, the docking time, the median operative time was significantly shorter for L-group: 130 (47) vs 180.38 (58) minutes (*p* < 0.001). Moreover, if we consider only SADI-S procedures, the median operative time was significantly shorter for L-group: 120 (49) vs 195 (48) min (*p* < 0.001).

**Table 3:** Data and statistical analysis of the laparoscopic-group and robotic-group after propensity score matching analysis

	Laparoscopic-group	Robotic-group	*p*
Patients	22	22	
Age (years)*	43.5 (15)	42.0 (13)	0.640
Height (cm)*	169.0 (14)	169.0 (19)	0.981
Weight (kg)*	153.0 (37.5)	158.5 (34.8)	0.745
BMI (kg/m^2^)°	54.5 (8.1)	53.4 (6.8)	0.690
Male/female	10/12	10/12	1
Smoking			
No	15	16	0.931
Previously (≥ 6 months)	5	4	
Previously (< 6 months)	2	2	
Comorbidities (yes/no)	16/6	12/10	0.347
HBP (yes/no)	8/14	7/15	1
OSAS (yes/no)	12/10	6//12	0.124
Diabetes			
No	16	18	
IGT	3	3	0.572
DMT2	3	1	
Previous non-bariatric abdominal surgery			
No	12	11	
Laparoscopic	2	3	0.885
Open	8	8	
Previous bariatric procedure			
No	21	21	1
Sleeve gastrectomy	1	1	
Procedure			
SADI-S	21	21	1
SADI	1	1	
Operative time (minutes)°	130.0 (47)	191.54 (49)	< 0.001
Docking time (minutes)	–	10.5 (8)	–
Intensive care unit (yes/no)	1/21	1/21	1
Postoperative hospital stay (days)°	3.0 (2)	2.0 (1)	0.062
Postoperative 30th day complications (yes/no)	0/22	3/19	0.233
Reoperation (yes/no)	0/22	1/21	1
Pneumonia (yes/no)	0//22	1/21	1
Bleeding (yes/no)	0/22	1/21	1
Trocar site hernia (yes/no)	0/22	1/21	1
30th day hospital readmissions (yes/no)	0/22	1/21	1
Late complications (yes/no)	1/21	0/20	1
Internal hernia (yes/no)	1/21	0/20	1

*BMI* body mass index, *HBP* high blood pressure, *OSAS* obstructive sleep apnea syndrome, *IGT* impaired glucose tolerance, *T2DM* type 2 diabetes mellitus, *SADI-S* single anastomosis duodeno-ileal bypass with sleeve gastrectomy, *SADI* single anastomosis duodeno-ileal bypass in previously sleeve gastrectomy

^*^Student’s *t* test

°Mann–Whitney *U* test

Furthermore, we reported 64 laparoscopic SADI-S, 30 laparoscopic SADI, 21 robotic SADI-S and one robotic SADI. The median operative times were 120 (38) min for laparoscopic SADI-S, 85 (55.25) min for laparoscopic SADI and 195 (47.5) min for robotic SADI-S. The operative time for laparoscopic SADI-S was longer compared to laparoscopic SADI (*p* < 0.001).

An analysis (CUSUM method) of all operative times of 3 type of procedure (laparoscopic SADI-S, robotic SADI-S, laparoscopic SADI) was performed by grouping patients chronologically into the respective groups.

A reduction in OT was observed after the first 47 cases for laparoscopic SADI-S (*p* = 0.090). A significant reduction in OT was observed after the first seven cases for robotic SADI-S (*p* = 0.023). No significant reduction was observed for laparoscopic SADI (*p* = 0.717).

Table 4 shows the distribution of the different procedures per years (laparoscopic vs robotic, primary versus revisional) during the study period.

**Table 4:** Distribution of the different procedures during the study period

Procedure	2016	2017	2018	2019	2020	2021
L-SADI-S	–	10	16	18	12	8
L-SADI	–	2	4	9	12	3
R-SADI-S	1	–	3	4	2	11
R-SADI	–	–	–	–	–	1

*L-SADI-S* primary laparoscopic procedure, *L-SADI* revisional laparoscopic procedure, *R-SADI-S* primary robotic procedure, *R-SADI* revisional robotic procedure

## Discussion

In this study, we present the results of our SADIS’ experience in an Italian high-volume bariatric center in terms of the outcomes of different surgical approaches. We performed the first SADIS’ case in July 2016, which was a robotic SADI-S, whereas the first laparoscopic SADI-S and the first laparoscopic SADI were performed in February 2017 and the only robotic SADI was performed in March 2021 [50]. Overall, the outcomes of 116 patients were reported. To our knowledge, this is the first monocentric Italian study on this procedure and the first case–control study on the outcomes of different surgical approaches (laparoscopic versus robotic) to perform this bariatric procedure.

In our experience, SADIS is a safe procedure with low postoperative complications, regardless of the surgical approach (laparoscopic versus robotic), with complication rates in line with other authors [5, 9, 25].

In our knowledge, this is the largest series of robotic SADIS procedures reported (22) since publications in this field are scarce [17–20]. Vilalonga et al. [19] reported three cases of robotic SADIS with mean operative time of 145 min. Martínez et al. [20] and Tarascò-Palomares et al. [18] reported two video cases: particularly in the second case, the operative time was 240 min of which 75 were necessary to repair an umbilical hernia. Finally, Tat et al. [17] described their experience of 12 robotic SADI-S, with a mean operative time of 204 min.

In our clinical experience, we prefer to use robotic approach in challenging cases, such as BMI ≥ 55 kg/m^2^, particularly in males, and/or with previously abdominal surgery. We believe that in this special population, the surgical approach with the robotic system will prove beneficial in relieving the surgeon of torque fatigue in patients with extra thick abdominal wall, which often results in leverage forces on trocar and instruments [26]. Furthermore, general reported advantages of robotic surgical systems over traditional laparoscopy include three-dimensional high-definition visualization, tremor filtration, direct camera control by the surgeon and wristed instruments, which make relatively complex laparoscopic tasks such as suturing easier. On this basis, more complex operations, that required two-step procedures, can be performed in a single-step operation easier and safely [27–30]. In the field of bariatric surgery, these characteristics translate, in example, into the ability to perform fully handsewn anastomoses versus the more commonly performed stapled anastomosis in laparoscopic surgery [31]. Multi-quadrant access is another benefit of recent robotic platforms [32]. During some type of bariatric surgery (gastric bypasses, biliopancreatic diversion, SADIS), the surgeon has to work in different abdominal quadrants to measure small bowel and perform anastomoses: unexpected findings will sometimes require to work in unplanned sections of the abdomen and this could determine the surgeon's decision to perform a different bariatric procedure not involving the small bowel (as sleeve gastrectomy) or even to abort the procedure [33]. Recent robotic platforms overcome this difficulty by allowing multi-quadrant access without the need to move or redock the platform [34] and this could potentially help the surgeon to successfully perform the initially planned procedure. Until recently, stapling task had to be performed by a bedside assistant during robotic surgery. The advent of robotic staplers has enabled the surgeon to position and fire stapler by himself: combined with the ability to control the camera and three different robotic arms at the console, robotic stapling can potentially obviate the need for a surgical trained bedside assistant during bariatric procedures [34]. Nevertheless, these technologies require not only surgeon’s but also operating room staff’s training [14] for time efficiency in robotic setting and docking [35].

To overcome potential selection bias and differences in groups’ size (laparoscopic and robotic), we used a propensity matching score analysis. After this randomization, the two groups (L-group and R-group) were comparable for demographic characteristics (as reported in Table 3) and particularly for preoperative BMI, which is higher in the L-group. To date, randomized controlled trial comparing laparoscopic and robotic SADIS (and similarly for BPD-DS) are lacking. The last highlights come from a retrospective study from Metabolic and Bariatric Surgery Accreditation and Quality Improvements Program (MBSAQIP) over 7235 minimally invasive procedures [36]. Our data reported statistically shorter operative time for L-group. This result is maintained even excluding the docking time for R-group and considering solely SADI-S procedures. We registered a median operative time for matched L-group of 130 min and 120 min for overall laparoscopic procedures. Other authors reported lower operative times [9], however, these data are not always available in all studies [5, 11, 37]. However, our series is heterogeneous for the surgical approach (laparoscopic vs robotic) and for the type of procedure (primary versus revisional) and this may influence the interpretations of these results. Furthermore, we perform only handsewn anastomoses and this differs from other authors who perform mechanical anastomoses: this could be another explanation of our longer operative time. Lastly, another important aspect which influences the operative time is the effect of the learning curve. As we reported in another study [38], we always consider the number of the laparoscopic and robotic bariatric procedures that have been performed by the surgeon or the surgeons. In this context, all the robotic and laparoscopic SADIS were performed by the same surgeon (M.R.), who performed more than 1000 laparoscopic bariatric procedures and more than 50 robotic bariatric procedures prior to performing the first robotic case (SADI-S), and in all cases, the duodeno-ileal anastomosis was handsewn in double layer. Schauer et al. and Buchs et al. reported that 100 cases are required to complete the learning curve for laparoscopic Roux-en-Y gastric bypass and 25–30 cases for the same procedure performed with robotic approach [39, 40], whereas Vilallonga et al. reported that 10 cases are needed for the learning curve of robotic sleeve gastrectomy [41]. Other authors reported that the learning curve from the laparoscopic approach to the robotic approach for the Roux-en-Y gastric bypass is challenging, requiring at least 75–100 procedures [40, 42]. Our analysis demonstrated a reduction of operative time for laparoscopic SADI-S after 47 cases and a significant reduction of operative time for robotic SADI-S after 7 cases but failed to identify a key point for the learning curves of the laparoscopic SADI. SADIS is a complex multi-quadrant procedure and so it is technically demanding. The extrapolation of previously reported data would lead to the conclusion that the more complex is the procedure, more cases are required to complete the learning curve and this seems to be in contrast with the obtained results. However, it should be kept in mind how the procedures have been carried out over time in our center (see Table 4): the 8th case of robotic SADI-S was performed after accomplishing 60 cases in total, therefore a cumulative transversal influence was probably determined between the different types of procedures. Indeed, the study of Surve et al. [9] reported a mean operative time of 67.2 min for 750 procedures, performed by 3 surgeons. Probably [43], a future analysis of our improved experienced with more cases will align with these data and will define the cut off for the learning curves. The median operative times for robotic approach are shorter than other anecdotical experiences: Tat et al. [17] reported an average median of 204 min on 11 case’s experience; Tarascó Palomares et al. [18] reported a single-case experience with a total operative time of 240 min (of which 165 min were required to complete the SADI-S procedure and 75 min to reduce and reparate an umbilical hernia) and Vilallonga et al. [41] experienced an average operative time of 145 min in 3 robotic SADI cases. Other experienced groups reported similar results with different bariatric procedures. Al-Mazrou et al. [36] reported longer mean operative time in robotic BDP-DS (219.2 vs 137.1; *p* < 0.001). Zhang et al. [44] in their meta-analysis showed that operative time was longer in robotic operations. A few studies reported the opposite [45, 46]. In particular, Ayloo et al. [45] reported a significantly longer mean operative time in laparoscopic group (227 vs 207 min).

In our study, we experienced more early complications in R-group, however, the re-operative complication rate was comparable and the overall complication rate in our series is low. Therefore, it is likely that this significance is attributable to the large number of the population study after randomization. In the comparative study of Al-Mazrou et al. [36] after multivariable analysis, there was no difference in overall complications, organ space infection, sepsis, or mortality between robotic and laparoscopic BPD-DS. Similarly, Zhang et al. [44] in their comprehensive systematic meta-analysis reported comparable leak, pulmonary embolism, estimated blood loss and stricture. Al-Mazrou et al. [36], not surprisingly, reported more deep thrombosis in robotic group, but this is probably attributable to its longer mean operative time. In our series, we did not experience this complication, probably because we extended the prophylaxis for 30 days after surgery. In fact, as Spaniolas et al. [47] reported in their study, this complication occurred at median time of 13 days, so we considered it correct to extend prophylaxis in these high-risk patients who underwent minimally invasive major abdominal surgery that required pneumoperitoneum for a long period of time. Interestingly, in our study, the median post-operative length of stay was almost significantly shorter in the robotic group. Zhang et al. [44] reported comparable length of stay for the two approaches and this is similar to the outcome reported by Al-Mazrou [36]. Different results in favor of the robotic approach were reported in other studies [16, 45]. However, it is possible that our results were partially influenced by the clinical experience over time. Indeed, the majority of robotic operations has been performed during the last 2 years of the study period, probably when we completed our “lesson learned” for the post-operative clinical management of these kind of patients and were more confident for a “fast-track” course after surgery. Precisely, to scientifically support this thesis, we utilized the CUSUM analysis to evaluate the learning curve of the post-operative clinical management for the discharge of these patients. These analysis shows the presence of a point of break at the 59th case, although not statistically significant (*p* = 0.136). The 59th case has been operated during 2019, which is before most of the robotic procedures have been performed.

The comparison of long-term nutritional complications is beyond the study outcomes, also due to the different median follow-up times.

The last aspect to analyze is the topic related to the costs, the analysis of which is not included the study outcomes. Nevertheless, we have gained experience in dealing with this topic in other research areas [48]. In our Health Care System, in which refund is based on the system of the “Diagnosis Related Groups” and which does not provide an additional reimbursement in case of robotic surgery, the use of this technology is partially limited to “small businesses”. In a period of spending review, the economic resources must be clearly weighted and, therefore, the use of economically disadvantageous technologies must necessarily find their own rationality. It is therefore important to identify cases where the use of robotic technology actually determines an advantage. Obviously, that is a difficult challenge. We used the robotic approach for challenging cases, but what is the meaning of “challenging cases” in our practice is not simple to explain. There is not a parameter, a nomogram or a characteristic that alone can define this condition. In our experience, “challenging cases” are patients with BMI ≥ 55 kg/m^2^, especially if males and/or with previously major abdominal surgery. In such a group of challenging patients, the robotic approach would facilitate the single step procedure. Since single step procedure seems to have a beneficial effect in terms of %EWL and comorbidity resolution [5, 6, 9]; the robotic approach could ultimately result in a better postoperative outcome and, probably, a reduction of the overall cost, eliminating the need for a second hospitalization and a second surgical procedure [49]. It is clear that such theoretical advantages should be explored and evaluated in larger comparative studies.

In our mind, the present study has the merit of being the first monocentric, case–control, comparative study for robotic and laparoscopic SADIS. However, it has several limitations that should be underlined. First of all, the present is a retrospective study over a long period of time. Second, the definition of the correct sample size was critical. In fact, if we consider a non-inferiority study for early complications (4%, in our overall analysis), with a significant level (α) of 5%, a power (1-β) of 95% and a non-inferiority limit (d) of 5%, the sample size required per group is 333 patients. Third, a cost-analysis was not performed.

In conclusion, SADIS is an effective bariatric and metabolic procedure with a low rate of early complications. Laparoscopic and robotic approaches seem to be comparable in term of safety for re-operative and post-operative complications, though the robotic procedures are burdened by longer operating times. Nevertheless, we trust that robotic approach may represent an added value in the most challenging cases, reducing the need for two-step procedures. However, larger studies with longer follow-up and cost-analysis are necessary to draw definitive conclusions.
